# Cardiovascular regeneration

**DOI:** 10.1186/scrt531

**Published:** 2014-12-17

**Authors:** Ronald A Li

**Affiliations:** Stem Cell and Regenerative Medicine Consortium; Department of Physiology, LKS Faculty of Medicine, University of Hong Kong, Pok Fu Lam, Hong Kong; Center of Cardiovascular Research, Icahn School of Medicine at Mount Sinai, New York, NY USA

## Abstract

Heart disease remains the number one cause of death in developed countries. Loss of cardiomyocytes (CMs) due to aging or pathophysiological conditions (for example, myocardial infarction) is generally considered irreversible, and can lead to lethal conditions from cardiac arrhythmias to heart failure. Human pluripotent stem cells (PSCs), including embryonic stem cells and induced pluripotent stem cells (iPSCs), can self-renew while maintaining their pluripotency to differentiate into all cell types, including CMs. As such, PSCs represent an unprecedented unlimited *ex vivo* cell source. In the present thematic series, we have solicited seven review articles to discuss the current state-of-the-art PSC-based approaches for such applications as disease modeling, discovery of novel drugs and therapeutics, cardiotoxicity screening and cell-based myocardial repair, as well as the associated hurdles and potential solutions.

In the first article by Jean-Sabastian Hulot and colleagues, the authors review a list of parameters that need to be considered for launching clinical trials of PSC-derived CMs [1]. These include the need for good manufacturing practice clinical grade cells, immunological consideration, an efficient cardiac differentiation protocol, purification of chamber-specific derived PSC-CMs, and optimized delivery methods for improved retention, survival and engraftment of the transplanted cells or tissues. The advantages and limitations of using large animal models such as porcine and non-human primates for pre-clinical testing are also discussed.

Hung-Fat Tse and Song-Yan Liao review the pros and cons of using multi-potent (adult) stem cells and PSCs for heart regeneration [2]. Specifically, human skeletal muscle myoblasts, bone marrow-derived cells, endothelial progenitor cells, mesenchymal stem cells and cardiac resident stem cells are compared with human embryonic stem cells and iPSCs. In brief, while adult stem cells represent a conceptually attractive autologous option, their ability to regenerate the myocardium appears to be limited.

While any stem cell-based clinical translations are going to take time because of defined regulatory processes that need to be followed, recent technological advances have enabled PSC-CMs to immediately serve as excellent *in vitro* diagnostic tools for drug discovery, toxicity screening and disease modeling. Arun Sharma, Sean Wu and Joseph Wu review the use of iPSCs for disease modeling and drug screening [3]. In their article, the LEOPARD syndrome, long QT syndromes, catecholaminergic polymorphic ventricular tachycardia, hereditary dilated and hypertrophic cardiomyopathy and arrhythmogenic right ventricular dysplasia/cardiomyopathy, which have been modeled using iPSCs, are given as specific examples.

Despite the promises of iPSC technology, however, there are hurdles that need to be overcome. A well-recognized roadblock has been the immaturity of PSC-CMs. The review article by Wendy Keung, Kenneth Boheler and Ronald Li summarizes the immature structural, electrophysiological, calcium-handling, bioenergetic and metabolic properties of PSC-CMs as well as our current understanding of their underlying molecular bases [4]. Based on this knowledge, *in vitro* approaches that focus on such developmental cues as neurohormones, epigenetic, micro-RNA and transcriptomic cues, and non-cell autonomous microenvironmental factors (for example, electrical and mechanical stimuli) have been accordingly developed to recreate niche environments for driving maturation. Along this line, Ken Boheler and colleagues [5] describe in their article how various physical parameters such as bio-scaffold, cell alignment and three-dimensional environments can contribute to the maturation and functionality of PSC-CMs.

Pragmatically, the use of PSC-CMs for *in vitro* diagnostics and/or cell-based therapies can be realized only if these cells can be mass produced with high standards of quality control. Steve Oh and colleagues [6] review considerations in designing bioreactor systems for scalable production of human PSC-CMs. Two-dimensional monolayer cell culture, cell-aggregate and microcarrier are compared in the context of platform selection, bioprocess parameters, medium development, downstream processing and parameters that meet current good manufacturing practice standards.

Although human PSC-based heart regeneration is a new concept and discipline with a relatively short history of about a decade, significant scientific and technical advances are being made at remarkably rapid rates, ushering in a new era of cardiovascular bio-engineering and regenerative medicine. It is with every optimism that the knowledge gained during the process not only enables a better basic understanding of the human heart but will also translate into tangible healthcare benefits in the not too distant future.

## Note

This article is part of a thematic series on *Cardiovascular regeneration* edited by Ronald Li. Other articles in the series can be found online at http://stemcellres.com/series/cardiovascular.

## Abbreviations

CM: Cardiomyocyte
iPSC: Induced pluripotent stem cell
PSC: Pluripotent stem cell.
